# Transcriptome analysis reveals key regulatory genes for root growth related to potassium utilization efficiency in rapeseed (*Brassica napus* L.)

**DOI:** 10.3389/fpls.2023.1194914

**Published:** 2023-07-19

**Authors:** Sani Ibrahim, Nazir Ahmad, Lieqiong Kuang, Keqi Li, Ze Tian, Salisu Bello Sadau, Sani Muhammad Tajo, Xinfa Wang, Hanzhong Wang, Xiaoling Dun

**Affiliations:** ^1^ Key Laboratory of Biology and Genetic Improvement of Oil Crops, Ministry of Agriculture and Rural Affairs, Oil Crops Research Institute of the Chinese Academy of Agricultural Sciences, Wuhan, China; ^2^ Department of Plant Biology, Faculty of Life Sciences, College of Natural and Pharmaceutical Sciences, Bayero University, Kano, Nigeria; ^3^ State Key Laboratory of Cotton Biology, Institute of Cotton Research, Chinese Academy of Agricultural Sciences (Institute of Cotton Research (ICR), CAAS), Anyang, China

**Keywords:** root, transcription factors, RNA-seq, DEGs, WGCNA, potassium utilization efficiency

## Abstract

Root system architecture (RSA) is the primary predictor of nutrient intake and significantly influences potassium utilization efficiency (KUE). Uncertainty persists regarding the genetic factors governing root growth in rapeseed. The root transcriptome analysis reveals the genetic basis driving crop root growth. In this study, RNA-seq was used to profile the overall transcriptome in the root tissue of 20 *Brassica napus* accessions with high and low KUE. 71,437 genes in the roots displayed variable expression profiles between the two contrasting genotype groups. The 212 genes that had varied expression levels between the high and low KUE lines were found using a pairwise comparison approach. The Gene Ontology (GO) and Kyoto Encyclopedia of Genes and Genomes (KEGG) functional classification analysis revealed that the DEGs implicated in hormone and signaling pathways, as well as glucose, lipid, and amino acid metabolism, were all differently regulated in the rapeseed root system. Additionally, we discovered 33 transcription factors (TFs) that control root development were differentially expressed. By combining differential expression analysis, weighted gene co-expression network analysis (WGCNA), and recent genome-wide association study (GWAS) results, four candidate genes were identified as essential hub genes. These potential genes were located fewer than 100 kb from the peak SNPs of QTL clusters, and it was hypothesized that they regulated the formation of the root system. Three of the four hub genes’ homologs—*BnaC04G0560400ZS*, *BnaC04G0560400ZS*, and *BnaA03G0073500ZS*—have been shown to control root development in earlier research. The information produced by our transcriptome profiling could be useful in revealing the molecular processes involved in the growth of rapeseed roots in response to KUE.

## Introduction

Root System Architecture (RSA), which encompasses more variations of cereal-based roots, describes the organization of the primary, lateral, and auxiliary roots on a wider scope. It is a key determinant of how efficiently plants utilize water and nutrients (Leitner et al., 2010; Tominaga-Wada et al., 2011; Zygalakis et al., 2011). The intrinsic nutritional condition of the plant influences systemic signaling in addition to local signaling that is induced by regional nutrient supply on the entire RSA (Giehl et al., 2014; Ohkubo et al., 2017). Studying RSA in cereals and biofuel crops is crucial since it can significantly impact plant yield and a plant’s capacity to flourish in “deficient” environments (Smith and de Smet, 2012). Additionally, altering a crop’s root system architectural traits to enable it to grow under drought or nutrient shortage circumstances can greatly increase the output (Wasson et al., 2012; Kuijken et al., 2015).

Transcriptome analysis has proven to be a successful method for finding candidate genes, and RNA sequencing of some crops has revealed a significant number of differentially expressed genes (DEGs) associated with root growth such as genome-wide expression patterns of the seedling primary roots in *B. napus* L. (Dun et al., 2016), Clubroot resistance in *B. rapa* L, (Wei et al., 2021), adaptive strategies to water deficit in *T. aestivum* (Mia et al., 2020), the effect of heat stress in seedling of *O. sativa* (Wang et al., 2022), effects of substrate and root hair formation in a spatial context in *Z. mays* (Ganther et al., 2021), the global gene expression profile of *H. vulgare* roots with varying capacities to withstand mild drought stress (Janiak et al., 2019). The broad comprehension of transcriptome control of root development in rapeseed is still unclear despite these transcriptomic investigations in several crops. Consequently, extensive study is required to fully understand the changes in the transcriptome and uncover the molecular basis of root development in rapeseed.

Weighted correlation network analysis (WGCNA), a system biology technique, is being used to evaluate gene relationship patterns between several samples. It can recognize groups of genes (modules) with comparable expression profiles, examine the connections between modules and sample phenotypes, depict the regulatory networks between the modules’ genes, and pinpoint key regulatory genes (Ackermann and Strimmer, 2009; Xiong et al., 2012). Three hub genes *GRMZM2G072121 (expansin precursor protein), GRMZM2G477658 (lipid-transfer protein), and GRMZM2G135536 (cytochrome P450 protein)* have been identified to be involved in the proliferation and elongation of cells, which may contribute to *Z. mays* root development (Hwang et al., 2018). Conversely, it was shown that *DcMYB113* controls the transportation of anthocyanins in *D. carota* roots (Xu et al., 2020). Transcriptome sequencing, genome-wide association study (GWAS), and WGCNA have been proven to be quick and effective methods for locating key candidate genes controlling root development (Que et al., 2018; Wang et al., 2019; Guo et al., 2020)

A widely grown plant in the *Brassica* genus called rapeseed (*Brassica napus*) is a significant source of vegetable oil that has historically been consumed by people. To assess RSA, optimize nutrient uptake, and maximize yield prospects for rapeseed, it is crucial to know the molecular pathways that influence root growth. This research was carried out to better understand how potassium utilization efficiency affects transcriptome changes and associated molecular mechanisms influencing rapeseed root development. In the current research, we employed RNA-seq to compare the root gene expression profiles of 20 *B. napus* lines with extremely low potassium efficiency to 20 lines with extremely high potassium efficiency at the seedling stage. We conducted a WGCNA assessment using these data to investigate the strongly correlated modules and co-expressed genes. More interestingly, this research might reveal candidate genes that would support molecular breeding and genetic engineering initiatives aimed at rapeseed root development enhancement.

## Materials and methods

### Plant materials and growing conditions

Using a hydroponic setup, the shoot fresh weight (SFW) of a *B. napus* association population with 327 accessions has been evaluated under control (6 mmol L^-1^ K) and K stress (0.01 mmol L^-1^ K), respectively (Li K. et al., 2021; Ibrahim et al., 2022). According to the differences in SFW and K utilization index (the ratio of SFW under K stress and CK treatment), 12 naturally occurring *B. napus* accessions with extremely high (S276, S229, S163, S115, S103, S235) and extremely low (S291, S208, S35, S41, S260, S112) SFW and 12 *B. napus* accessions with extremely high (S191, S221, S103, S208, S276, S20) and extremely low (S91, S232, S112, S32, S315, S160) K utilization index were chosen. These accessions were primarily categorized into four groups, named Group HK1, LK1, HK2, and LK2 based on the phenotypic parameters, respectively. According to a prior Ibrahim et al. (2022) report, plants of the designated *B. napus* accessions were hydroponically grown in a greenhouse under two K conditions, low K level (LK, 0.01 mM K^+^) and high K level (HK, 6 mM K^+^). Three plants out of each accession were collected after growth for 7 and 14 days at the two different time points (T1 and T2). The SFW was evaluated to assess how a potassium deficit might affect rapeseed development. Root samples were selected for transcriptome analysis based on SFW analysis.

### RNA sequencing

Root tissues of the designated *B. napus* accession in each group were mixed equally. From the root tissue of four groups with high-K and low-K efficiencies, total RNA was isolated. For RNA sequencing (RNA-seq), three biological replicates from three different plants were collected for each sample. A total of 48 RNA-seq libraries (one tissue × four groups × two treatments × two-time points × three biological replicates per sample) were produced for total RNA extraction using the IRIzol reagent. The sequencing library was constructed and sequenced on an Illumina HiSeqTM 2500 platform by the Oebiotech Company in Shanghai, China. A filter and alignment were applied to raw reads with 150 paired-end base pairs (bp), as previously described by Ahmad et al. (2022).

### Sequence alignment to the *B. napus* reference genome and RNA-sequencing data analysis

Clean reads were produced after sequencing by eliminating adaptor sequences, poor-quality reads, and unclear bases (N). After trimming and filtering, the descriptive statistics for the clean data, including Q20, Q30, GC content, and sequence repetition level, were also computed at the same time. The subsequent analysis used the clean reads after that. The clean reads were mapped to the *B. napus* ZS11 reference genome (http://www.genoscope.cns.fr/brassica napus/data/) using Hisat2. The log2 fold change (FC) between the genes from the lines with extremely high and low potassium efficiency was employed to identify the genes that were differentially expressed. DEGs with |log2 (FC)| ≥ 2 and statistical significance *P* ≤ 0.05 were chosen. The “DESeq” tool in R was used to find DEGs using the criteria |log2 ratio| ≥ 1 and *P* ≤ 0.05 for false discovery rate (FDR).

### WGCNA

WGCNA may be used to identify hub genes with significant impacts and modules of highly connected genes (Li K. et al., 2021). WGCNA was used to apply all DEGs between the HK and LK groups in paired comparisons with *P* < 0.05. In sixteen separate groups, the correlation between co-expressed genes was evaluated. Using R’s WGCNA package, the WGCNA was carried out (Langfelder and Horvath, 2008). We used |log2 ratio| ≥ 1 and a false discovery rate (FDR) of *P* ≤ 0.05 to find DEGs using the “DESeq” R package.

### Gene set enrichment analyses (GSEA)

GSEA is an effective statistical technique for detecting overrepresented genes, classifying them, and scoring them based on their false discovery rate (FDR) p-value (Howe et al., 2015). We examined the genes whose differential expression was identified in two separate comparisons. Without initially determining if they were increased in the first or second assessment, we first selected the gene sets in both comparisons. Using the Oebiotech website, the DEGs’ gene ontology (GO) and Kyoto Encyclopedia of Genes and Genomes (KEGG) pathway enrichment analyses were performed (https://cloud.oebiotech.cn/task/). Except for the gene set false discovery rate (FDR) p-value limit of *P* ≤ 0.05, the parameters were set as described by Howe et al. (2015). The biological process, cellular components, and molecular functions datasets from Gene Ontology (GO) were the three we examined.

### Validation of DEGs by qRT-PCR

Nine genes arbitrarily chosen from the DEGs were subjected to a quantitative real-time PCR (qRT-PCR) to validate the accuracy of the RNA-seq data. SYBR qPCR Master Mix (Vazyme) was employed with the CFX96 for qRT-PCR analysis (BIO-RAD). Each sample was subjected to three technical replications. The *B. napus* ACTIN2 was used as an internal control in the 2^−ΔΔCT^ method to calculate the relative expression of the target genes.

## Results

### Differentially expressed genes (DEGs) between high and low potassium efficient groups

In previous studies by Ibrahim et al. (2022), the results of the hydroponic screening of accessions for K stress were reported. Twelve *B. napus* accessions with extremely high or low shoot biomass weight and twelve *B. napus* accessions with extremely high or low K utilization index were chosen and mixed into four groups. These groups were given the names group1-HK1, group1-LK1, and group2-HK2 and group2-LK2, respectively, based on the K utilization index (the ratio of shoot biomass weight under low k and CK treatments) and the SFW (shoot biomass weight as determined by the association panel). **Figure S1A** demonstrates that the SFW of group-HK1 was significantly greater than group-LK1 at both two-time points, T1 (7 days after transplantation) and T2 (14 days after transplantation) and that group1-HK1 and group1-LK1’s SFW under the high level were significantly larger than those under the low level. When compared to group-2 LK2, the K efficiency index STI-K of group-2 HK2 was much higher (**Figure S1B**). By doing this, the samples are guaranteed to be of high enough quality for the subsequent transcriptome analysis.

According to the two sampling points, two treatments, and four groups of high and low potassium efficiency materials, a total of 48 libraries were generated, including three biological replicates of HK and LK groups under low potassium stress and control conditions. After trimming and filtering, the Illumina RNA-Seq analysis of the 48 samples yielded 316.33G of clean data and 2,305,858,668 total reads. The average GC content was 47.32%, while the Q30 was approximately 94.04% (**Table S1**). The sequence was aligned with the specified *B. napus* reference genome after the reads were processed, yielding 2305.86 million clean reads (**Table S2**). The several map ratios varied from 8.83% to 9.99%, while the distinct map ratio varied from 84.11% to 86.77% (**Table S2**).

**Figure 1:**
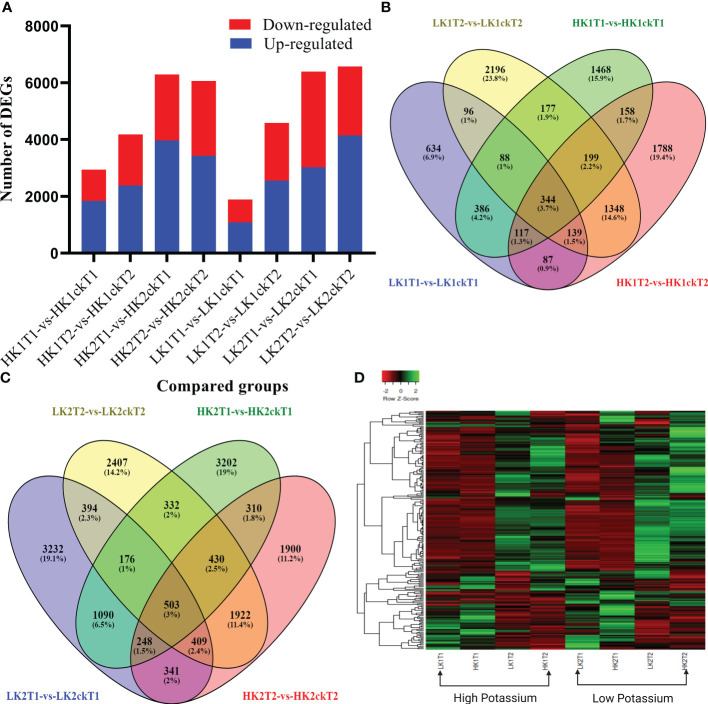
Analyses of differentially expressed genes (DEGs). **(A, B)** A Venn diagram showing the DEGs between the low and high efficient groups respectively. **(C)** Summaries of differentially regulated genes during the time course (T1 and T2). **(D)** Heatmap showing the Hierarchical cluster analysis of 202 common DEGs between the high and low K absorption efficient group.

To identify which genes were essential in each group, we analyzed DEGs between the corresponding materials in the two potassium efficiency groups, respectively at both time points (7DAT and 14DAT). To compare the DEGs between the two categories, the false discovery rate (FDR) ≤ 0.05 and the absolute value of |log2 (fold change) | ≥ 1 were employed as criteria. In total, 2,937, 4,180, 1,891, and 4,587 DEGs were identified (**Table 1**) in HK1T1-vs-HK1ckT1, HK1T2-vs-HK1ckT2, LK1T1-vs-LK1ckT1, and LK1T2-vs-LK1ckT2 (1,840, 2,384, 1,084, and 2,554 upregulated and 1,097, 1,796, 807, and 2,033 downregulated; **Figure 1A**). 6,291, 6,063, 6,393, and 6,573 DEGs were identified in HK2T1-vs-HK2ckT1, HK2T2-vs-HK2ckT2, LK2T1-vs-LK2ckT1, and LK2T2-vs-LK2ckT2 (3,969, 3,427, 3,022, and 4,143 upregulated and 2,322, 2,636, 3,371, and 2,430 downregulated; **Figure 1A**). For two independent comparison groups, the 344 and 503 common DEGs, (HK1T1-vs-HK1ckT1, HK1T2-vs-HK1ckT2, LK1T1-vs-LK1ckT1, and LK1T2-vs-LK1ckT2) and (HK2T1-vs-HK2ckT1, HK2T2-vs-HK2ckT2, LK2T1-vs-LK2ckT1, LK2T2-vs-LK2ckT2) were identified (**Figures 1B, C**; **Tables S3**, **S4**). Additionally, 212 DEGs were common in both groups utilizing a pairwise comparison method, and a hierarchical cluster analysis was performed on them (**Figure 1D**). Of these, 132 were upregulated and 80 were downregulated (**Table S5**).

**Table 1:** The differentially expressed genes (DEGs) number between eight samples using (P-value < 0.05&|log2FC|>1) as the significant cutoff.

DEG set name	Number of DEG	Up-regulated	Down-regulated
HK1T1-vs-HK1ckT1	2,937	1,840	1,097
HK1T2-vs-HK1ckT2	4,180	2,384	1,796
HK2T1-vs-HK2ckT1	6,291	3,969	2,322
HK2T2-vs-HK2ckT2	6,063	3,427	2,636
LK1T1-vs-LK1ckT1	1,891	1,084	807
LK1T2-vs-LK1ckT2	4,587	2,554	2,033
LK2T1-vs-LK2ckT1	6,393	3,022	3,371
LK2T2-vs-LK2ckT2	6,573	4,143	2,430

### GO and KEGG analysis of the DEGs

**Figure 2:**
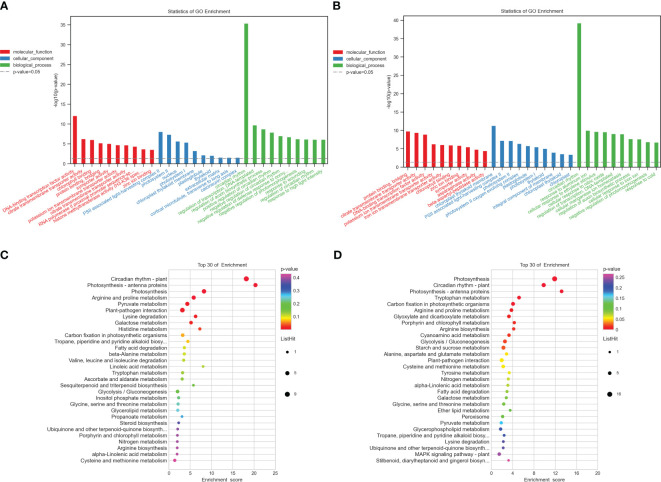
Functional annotation of all the commonly detected DEGs in the two groups. **(A, B)** Gene Ontology (GO) analyses of commonly differentially expressed genes in the two groups at two-time points (T1 and T2). **(C, D)** Kyoto Encyclopedia of Genes and Genomes (KEGG) analysis of commonly differentially expressed genes in the two groups at two-time points (T1, T2), respectively. −log_10_ (*p*-value) is the Y-axis. As the height of the bar chart rises, the pertinent p-value falls. The hues red, blue, and green denote molecular functions, cellular components, and biological processes, respectively.

To pinpoint the crucial phase transition-associated genes, the 344 and 503 identical DEGs were submitted to an enrichment analysis for GO annotation terms. **Tables S6**, **S7** display the top 20 significantly enriched GO terms. The biological process, cellular component, and molecular function GO categories were all well underrepresented in the first group. The most widely used GO terms are depicted in **Figures 2A, B**. In the category of molecular function, chlorophyll-binding was followed by citrate transmembrane transporter activity as the most often occurring GO term. The most abundant cellular component was PSII-associated light-harvesting complex II, followed by photosystem II and the nucleus. The circadian rhythm, transcription regulation, DNA-templated, and auxin biosynthetic process regulation were the most abundant biological processes, in that order. The majority of GO terms in the category of molecular function, however, were related to protein binding and bridging, citrate transmembrane transporter activity, and DNA-binding transcription factor activity. The light-harvesting complex II associated with PSII and photosystem II was the next most frequent cellular component, followed by the chloroplast thylakoid membrane. The circadian rhythm was the most prevalent biological function, followed by aluminum ion response and cellular response to ethylene stimulation in the group with high potassium efficiency. Particularly, several genes were classified into multiple categories.

Additionally, to comprehend the metabolic pathways in each of the two groups, we performed a KEGG enrichment analysis (**Figures 2C, D**). Arginine and proline metabolism (bna00330), photosynthesis, photosynthesis-antenna proteins (bna00196), and circadian rhythm-plant (bna04712) were discovered to be considerably enriched in both groups and each one of them has a role in the development and expansion of plant roots (**Tables S8**, **S9**).

### Identification of root development-related genes

The DEGs from the two categories were subjected to the VENN analysis to investigate the genetic influences on root development (**Figures 1B, C**). Between the groups with high potassium absorption efficiency and low potassium absorption efficiency, 212 DEGs were discovered to be overlapped (**Table S5**). These included *BnaA02G0069100ZS* RVE 1, *BnaA05G0006700ZS* GOLS 1, *BnaA10G0008600ZS* LHY, and *BnaA03G0311700ZS* DTX43 homologous to the Arabidopsis *Protein REVEILLE 1 (RVE1*), *Galactinol synthase 1* (*GOLS1*), *Late elongated hypocotyl* (*LHY*), and *Protein DETOXIFICATION 43* (*DTX43*) were implicated in the cellular reactions to ethylene, gibberellin, abscisic acid, and auxin-activated signaling, all of which have been documented to affect root growth (Rahman et al., 2007; Tian et al., 2009; Ubeda-Tomás et al., 2009; Luo et al., 2014), were discovered to be stridently expressed.

### Transcription factor (TF) encoding and hormone-linked to root genes

Under certain environmental conditions, transcription factors are essential as molecular switches that regulate the expression of specific genes and influence plant growth and development (Mia et al., 2020). In this study, DEGs from both categories that encode transcription factors (TFs) were found to be 33 (**Table S10**). Eight members of the ethylene-responsive transcription factor (ERF), five of the heat stress transcription factor A (HSFA), three of the LUX transcription factor, three of the MYB transcription factor, two of the basic helix-loop-helix (bHLH) transcription factor, and one each of the WRKY and ABR 1 transcription factor was among the 33 transcription factors encoding DEGs (**Figure S2A**). DEGs linked to hormones and signaling pathways, however, have their expression profiles analyzed to more fully comprehend signaling amid phenotypic variations in *B. napus* root traits. We identified 6, 15, 6, 24, and 6 genes, respectively, in the two groups that function in the SA, Auxin, GA, ABA, and JA signaling pathways. While over 65% of the genes involved in ABA signaling were downregulated, over 90% of the genes involved in auxin and GA signaling were upregulated (**Figures S2B–F**).

### Carbohydrate, lipid, and amino acid metabolism-related DEGs

To alter the root architecture and absorb enough nutrients and water, roots multiply. This has altered the expression of genes involved in lipid metabolism, cell wall production, and carbohydrate metabolism (Rasheed et al., 2016). To determine if the molecular and biochemical levels of carbohydrate, lipid, and amino acid metabolism were significantly different between the high and low K absorption efficiency groups, we carefully examined the pathways of the associated genes (**Tables S8, 9**). There were differences in the expression of genes related to carbohydrate metabolism during galactose metabolism, starch, and sucrose metabolism, pyruvate metabolism, inositol phosphate metabolism, and pentose and glucuronate interconversions, among other processes. Three genes related to glycolysis/gluconeogenesis, three genes related to galactose metabolism, two genes related to inositol phosphate metabolism, one gene related to starch and sucrose metabolism, five genes related to pyruvate metabolism, and one gene related to pentose and glucuronate interconversions all had changed expression, suggesting that energy and carbon consumption blocks are indeed required. Two of the three genes linked to glycolysis/gluconeogenesis that were upregulated were *BnaC01G0229100ZS* (*L-LDH*) and *BnaA01G0039400ZS* (*ALDH3I1*), whereas *BnaA03G0539900ZS* was downregulated (**Table S3**). Missihoun et al. (2018) found that the loss of *ALDH3I1* and *ALDH7B4* function led to changes in glutathione pools, NAD(P)H levels, and the NAD(P)H/NAD(P) ratio. Additionally, a GSH-dependent developmental route that is necessary for cell division to begin and continue during postembryonic root growth has been identified (Vernoux et al., 2000).

Lipid metabolism, a highly coordinated process, includes fatty acid (FA) production, fatty acid (FA) elongation, and lipid degradation (Troncoso-Ponce et al., 2011). Four genes involved in glycerophospholipid metabolism were expressed differently in the group with low K absorption efficiency: *BnaC03G0307400ZS*, *BnaC03G0307200ZS* encoding *PLDGAMMA1*, and *BnaA01G0154700ZS* (*PSD3*) were downregulated, whereas *BnaC09G0308400ZS*, which encodes *CEK3*, was upregulated (**Table S9**). *Arabidopsis thaliana CEK3*, a Cho-specific kinase, is connected to cell elongation during root development. Through phylogenetic investigation of *CEK* orthologs in Brassicaceae species, it was demonstrated that Etn kinases and Cho kinases have unique evolutionary histories (Lin et al., 2020).

Single amino acids like glutamine, amides, peptides, or mixtures of ON compounds were found to cause root elongation, adventitious root formation, and an increase in the root-to-shoot ratio. Conversely, high concentrations of single amino acids like glycine nearly completely inhibited root growth (Gruffman et al., 2012). *BnaA08G0131200ZS*, *BnaC03G0740800ZS*, *BnaC01G0037500ZS*, which encode *CAT2*, and *BnaA02G0068000ZS*, which encodes *NIT2*, are the four DEGs that were considerably upregulated in the low K efficient group (**Table S9**). In addition, the signaling pathways or composition of numerous phytohormones are altered by the oxidative stress caused by the loss of *CAT2* activity (Chaouch et al., 2010; Han et al., 2013). All cat2-containing lines experienced lower root growth, but this inhibition was reversed when plants were grown in high CO_2_, suggesting that stress at the leaf level has a more indirect effect (Yang et al., 2019). The DEGs in the low K efficient group that encode *ADC2*, *POX1*, and *SAMDC3* were upregulated, and they are *BnaC07G0522700ZS*, *BnaA02G0358100ZS*, and *BnaA01G0415300ZS* respectively (**Table S9**). The model plant, *Arabidopsis thaliana*, seems to only have the arginine decarboxylase pathway. It has two paralogs of arginine decarboxylase (*ADC1* and *ADC2*). As a result, the significance of transcriptional modulation in the expression of the *ARGININE DECARBOXYLASE* (*ADC2*) gene was highlighted. Variations in promoter activity were related to changes in mRNA levels in seed germination, root growth, and response to chilling (Hummel et al., 2004).

### DEGs associated with hormonal metabolisms/signaling pathways during root development in *Brassica napus*

To better comprehend signaling across the developmental stages of the *B. napus* root, we concentrated on the expression profiles of DEGs implicated in hormone and signaling pathways. Auxin regulates cell division, differentiation, and elongation during root growth. Recent research has shown that key plant hormones like auxin, abscisic acid, brassinosteroids, gibberellic acid, cytokinin, and ethylene play a significant role in the production and dispersion of primary root development.

There were changes in the expression of four auxin-responsive genes in both low and high-K efficient groups, including three upregulated DEGs (*BnaA07G0023000ZS*, *BnaC07G0044800ZS*, and *BnaC04G0012800ZS*) and one downregulated DEG (*BnaA01G0364500ZS*) that encode SAUR-like auxin-responsive protein family members (**Table S8**). By regulating auxin transport, SAUR proteins aid in the development and proliferation of cells (Kong et al., 2013). Reduced rice root length is caused by overexpression of *SAUR39* (Kant et al., 2009). In conjunction with gene expression patterns, *BnaA07G0023000ZS, BnaC07G0044800ZS, BnaC04G0012800ZS*, and *BnaA01G0364500ZS* may control root growth by limiting cell expansion/proliferation in the root.

Several phytohormones, primarily through interaction with auxin or among themselves, such as cytokinins, abscisic acid, ethylene, gibberellins, and brassinosteroids, play significant roles in root growth (De Smet et al., 2015). Salicylic acid, abscisic acid, gibberellin, and jasmonic acid have each been connected to 24, 6, 6, and 6 DEGs, respectively (**Figures S2B–F**). *BnaA05G0363900ZS, BnaA09G0025200ZS*, and *BnaC04G0011300ZS*, three DEGs associated with abscisic acid metabolism, were homologous to *AtCLP5, AtSAG21*, and *AtATHB-12* (**Table S6**). However, *AtCLP5* is a special member of the *CLP* family found in Arabidopsis, which positively regulates drought responses and ABA-mediated development (Jin et al., 2011). In plants, ABA performs several crucial roles in a range of processes, such as embryogenesis, seed maturation, dormancy, root and shoot growth, and so forth (Zhang et al., 2006). Additionally, the DEGs *BnaA10G0008600ZS, BnaC03G0001300ZS*, and *BnaC05G0010100ZS* are upregulated in the response to gibberellin encoding *LHY* (**Table S7**). As an organ of the plant specifically adapted for symbiotic nitrogen transfer between microsymbiont and host, nitrogen-fixing root nodules have evolved in legume species. *Medicago truncatula* nodules’ endogenous circadian rhythm is controlled by *MtLHY*. As a result of *MtLHY’s* loss of function, fewer nodules are produced, which reduces the capacity to uptake nitrogen (Kong et al., 2020).

### Real-time PCR validation of DEGs

**Figure 3:**
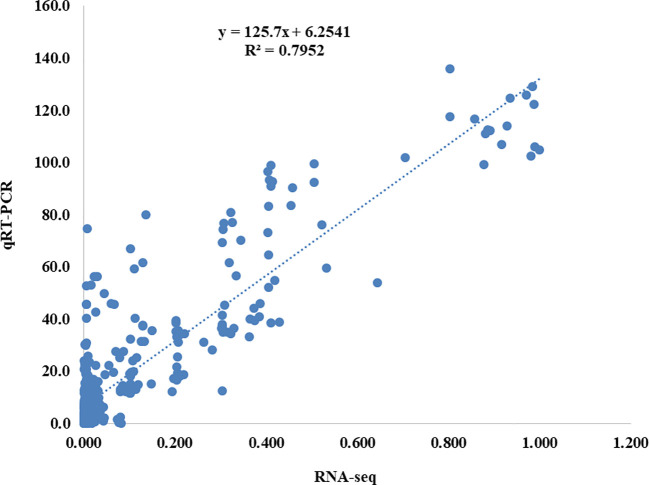
Correlation analysis between the results of qRT-PCR and RNA-seq results. The y-axis shows the qRT-PCR analysis results, while the x-axis shows the RNA-seq data.

All of the samples were subjected to qRT-PCR for nine genes to confirm the outcomes of the RNA-Seq analysis. The RNA-Seq data was reputable because the qRT-PCR results were highly congruent with them (**Figure 3**). Provided here were the primer sequences and the *B. napus ACTIN2* gene-specific primer (**Table S11**).

### Gene co-expression modules associated with root-related traits

**Figure 4:**
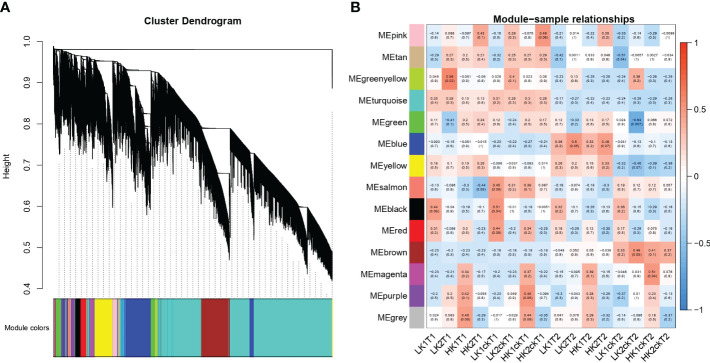
WGCNA of gene expression matrix in rapeseed. **(A)** a Hierarchical clustering tree (dendrogram) of genes based on co-expression network analysis. The color on the X-axis represents the module colors. **(B)** Module–sample association. Each row corresponds to a module labeled with a color identical to that in b, and each column corresponds to a sample. Correlations between the various modules are represented by the values inside the colored boxes.

To discover co-expression gene modules, 49,080 DEGs from the 16 root samples were utilized in a weighted gene co-expression network analysis (WGCNA) on all 16 root RNA-Seq data. According to the gene correlations, a total of 14 modules were found in the dendrogram (**Figure 4A**), and the correlations between modules and samples were shown in (**Figure 4B**). Remarkably, we discovered that LK1ckT1 was substantially related to three modules: salmon, black, and red. Moreover, we found that the green-yellow module correlates more strongly with LK2T1, the blue module with LK2T2 and HK2T2, and the magenta module with HK1ckT2, respectively. Similar to how the brown module and LK2ckT2 were substantially correlated, the pink module and HK2T1 and HK2ckT1 were as well, while HK1ckT1 and HK1T1 showed a substantial correlation with purple and grey modules, respectively. Nevertheless, gene ontology analysis revealed that genes in the green-yellow, magenta, and blue modules were highly significantly enriched (*P* < 0.05) and involved in the GDP-L-fucose salvage, positive regulation of cellular response to amino acid starvation, and photosynthesis (**Tables S12–S14**; **Figures S3A–C**).

We combined the results of our DEGs and WGCNA with our previous GWAS results (Ibrahim et al., 2022). According to GWAS data, 1,360 genes were discovered in the haplotype blocks of the 45 QTN clusters. 13 potential genes overlapped between GWAS and DEGs, and 7 potential genes overlapped between GWAS and WGCNA when the moderate correlation of WGCNA genes in the module (r^2^ > 0.50) was taken into account (**Table 2**). Additionally, 4 genes showed overlap in GWAS, DEGs, and WGCNA data (**Table 2**). Three of the seven GWAS/WGCNA overlapped genes were expressed more highly across all samples in the blue, brown, and magenta modules, as were two genes in the yellow modules and one gene in the brown module. In every sample, overlapping genes were highly expressed. Five genes in the blue module were situated within less than 100 kb from the lead SNPs, namely *BnaA01G0344100ZS, BnaA02G0216100ZS, BnaA01G0343300ZS, BnaA01G0343400ZS*, and *BnaA04G0189000ZS*, which were positioned 13.51 Kb, 42.73 Kb, 53.51 Kb, 49.10 Kb, and 54.64 Kb from the lead SNPs of *qRT.A01-1, qRT.A02-4, qRT.A01-1*, and *qRT.A04-3* (**Table 2**). *Pyridoxine biosynthesis 1.2* genes encoded by *BnaA01G0344100ZS* impacts root growth by controlling Arabidopsis LR initiation under cold stress (Jeon et al., 2016). A strong association was found between B*naA05G0032700ZS* and four genes, *BnaC06G0226000ZS, BnaC02G0500800ZS*, BnaC01G0183000ZS, and BnaA09G0485400ZS in the green-yellow module (**Figure S4A**). A homolog of *BnaA05G0032700ZS* identified as *tetratricopeptide-repeat thioredoxin-like 3 (TTL3)* interfaces with microtubules and may be a link between cytoskeletal function and the brassinosteroid signaling pathway in the formation of lateral roots (Xin et al., 2020).

**Table 2:** Differentially expressed genes (DEGs) identified by GWAS, WGCNA, and differential expression analysis.

QTN Cluster	Gene ID	Module	Distance to lead SNP (Kb)	A.T Homolog	Gene symbol	Description
*qRT.A01-1*	^a^BnaA01G0344100ZS	Turquoise	13.51	AT3G16050.1	*PDX1.2*	Pyridoxine biosynthesis 1.2
*qRT.A02-11*	^a^BnaA02G0140000ZS	Turquoise	-94.04	AT5G53290.1	*CRF3*	Cytokinin response factor 3
*qRT.A02-4*	^a^BnaA02G0216100ZS	Turquoise	42.73	AT1G75520.1	*SRS5*	SHI-related sequence 5
*qRT.A04-5*	^a^BnaA04G0154400ZS	Turquoise	-58.99	AT2G23460.1	*XLG1*	Extra-large G-protein 1
*qRT.A05-11*	^b^BnaA05G0049000ZS	Turquoise	94.97	AT2G44920.2		Thylakoid lumenal 15 kDa protein 1
*qRT.A07-6*	^a^BnaA07G0119000ZS	Turquoise	25.95	AT1G23080.3	*PIN7*	PIN-FORMED 7
*qRT.A07-1*	^a^BnaA07G0370900ZS	Yellow	7.37	AT1G77920.1	*BZIP*	BZIP transcription factor family protein
*qRT.A08-3*	^a^BnaA08G0184400ZS	Turquoise	78.15	AT4G36760.1	*APP1*	Aminopeptidase P1
*qRT.A01-8*	^b^BnaA01G0101300ZS	Greenyellow	-98.47	–		
	^b^BnaA01G0098300ZS	Turquoise	67.87	AT4G18810.2		Probable complex I intermediate-associated protein 30
*qRT.A01-1*	^b^BnaA01G0343300ZS	Blue	53.51	AT3G16150.1	*ASPGB1*	ASPARAGINASE B1
	^b^BnaA01G0343400ZS	Blue	49.10	AT3G16140.1	*PSAH1*	Photosystem I reaction center subunit VI-1
*qRT.A07-1*	^b^BnaA07G0372400ZS	Brown	92.35	AT1G78090.1	*TPPB*	Trehalose-phosphate phosphatase B
	^b^BnaA07G0369600ZS	Salmon	82.02	AT1G77640.1	*ERF012*	Ethylene-responsive transcription factor
*qRT.A09-14*	^b^BnaA09G0056600ZS	Yellow	91.44	AT5G27540.2	*MIRO1*	Mitochondrial Rho GTPase 1
*qRT.C04-2*	^c^BnaC04G0560400ZS	Yellow	23.19	AT2G38170.1	*CAX1*	Vacuolar cation/protein exchanger 1
*qRT.C08-1*	^b^BnaC08G0503100ZS	Blue	94.58	AT1G12900.1	*GAPA2*	Glyceraldehyde-3-phosphate dehydrogenase
*qRT.A03-17*	^c^BnaA03G0073500ZS	Blue	80.37	AT5G17300.1	*RVE1*	Protein REVEILLE 1
*qRT.A04-3*	^c^BnaA04G0189000ZS	Blue	54.64	AT2G29320.1		NAD(P)-binding Rossmann-fold superfamily protein
*qRT.A10-1*	^c^BnaA10G0258200ZS	Blue	89.71	AT5G06980.4	*LNK4*	NIGHT LIGHT-INDUCIBLE AND CLOCK-REGULATED GENE 4

^a^DEGs overlapped by GWAS and WGCNA; ^b^DEGs overlapped by GWAS and differential expression analysis; ^c^DEGs overlapped by GWAS, WGCNA, and differential expression analysis.

Moreover, the green-yellow module demonstrated strong correlations between five DEGs, *BnaA02G0236100ZS, BnaA02G0265300ZS, BnaA08G0297500ZS, BnaC03G0674900ZS*, and *BnaC06G0455000ZS* (**Figure S4A**). Six DEGs in the magenta module, *BnaA03G0266800ZS, BnaA04G0257700ZS, BnaA05G0411900ZS, BnaA09G0711700ZS*, and *BnaC06G0365400ZS*, had a strong association to one another (**Figure S4B**). *Protein phosphatase 2A* (*PP2A*), a homolog of *BnaC06G0365400ZS*, was discovered to be a positive regulator of auxin redistribution caused by osmotic stress-induced root system architecture alteration in *Arabidopsis thaliana* (Chávez-Avilés et al., 2013).

## Discussion

Root development is a crucial characteristic that can affect crop output and quality. Determining the genetic loci for root development features may be useful for understanding the genetic basis of root growth and for creating cultivars that are adaptable to various environments. Due to its great resolution and lower cost compared to microarray analysis, RNA sequencing has been widely used to analyze variations in overall gene expression. Nonetheless, a significant number of differentially expressed genes (DEGs) amongst samples were frequently discovered by transcriptome analysis (Wang et al., 2020). In this study, we used RNA-Seq to investigate the relationship between transcriptional profiles and the root system architectural development between high and low-potassium-efficient groups of *B. napus*. We have identified 344 and 503 common DEGs specific to groups with high K efficiency and group with low potassium efficiency, while 212 DEGs were considered common DEGs for both groups. Moreover, distinct genotypes with different rates of potassium absorption were studied for the transcriptome changes in rapeseed roots during early seedling growth. The idea was to examine the underlying mechanism and uncover the key genes, transcription factors, and complicated pathways that are essential for root development in rapeseed during the early growth stage by coupling these changes with variations in gene expression between the contrasting genotypes. The annotated transcripts revealed 12,976 DEGs at a time point (T2) and 9,915 DEGs at a time point (T1), respectively, indicating that the majority of the DEGs were up-regulated at the latter time point and down-regulated at the former (T1). These findings imply that the differential gene expression at a certain time point (T2) may be more crucial to root growth than the gene expression at a different time point (T1).

DEGs linked to significant GO terms may be crucial for root development and stress tolerance/potassium efficacy. *BnaA02G0271600ZS* (*AtHKT1*), for instance, is linked to GO-term potassium ion transport. The ability of plants to ensure high K^+^ concentrations under salt stress is another useful tactic (Mäser et al., 2001). Several plant species, including the model plant Arabidopsis, rice, sorghum, and tomato, as well as extremophile models like *Eutrema parvula*, have been used to study the crucial role of *HKT1* transporters under salt stress (Uozumi et al., 2000; Golldack et al., 2002; Asins et al., 2013; Wang et al., 2014; Ali et al., 2018).

The intensity and specificity required to find DE candidate genes can be obtained by combining genome-wide association study (GWAS) with RNA-seq, and this approach is more effective than each method by itself (Zhang et al., 2015; Hua et al., 2016; Lu et al., 2016). In *B. napus*, 8, 24, and 174 candidate genes influencing traits related to the harvest index, stem resistance to *Sclerotinia sclerotiorum*, and drought tolerance were discovered based on unified comparisons of RNA-seq and GWAS. More subsequently, a *NODULIN 26-LIKE INTRINSIC PROTEIN* gene involved in controlling boron uptake efficiency in *B. napus* was discovered using a combination of whole-genome re-sequencing (WGS), digital gene expression (DGE) profiling, and QTL fine mapping (Hua et al., 2016). Similar to the previous study, 13 potential genes that have been demonstrated to act as regulators of Arabidopsis root growth were identified by sequencing the transcriptomes of high potassium efficiency (HK) and low potassium efficiency (LK) lines and comparing the DEGs with GWAS. For instance, the Arabidopsis plastidial (*GAPCps*) *GAPDH* plays a crucial role in the regulation of primary metabolism in plants. A lack of *GAPCp* impacts the metabolism of amino acids and sugars and hinders plant growth. Serine availability to roots is particularly impacted by *GAPCp* deficiency, which has a striking phenotype of delayed root development (Muñoz-Bertomeu et al., 2010). The candidate gene *BnaA10G0258200ZS*, which is similar to NIGHT *LIGHT-INDUCIBLE AND CLOCK-REGULATED GENE 4* (*LNK4*), participates in the response to karrikin. According to Swarbreck et al. (2019), root skewing was altered in *Arabidopsis thaliana* when mutations in the MAX2 *(MORE AXILLARY GROWTH 2*) F-box leucine-rich protein and the *KAI2* (*KARRIKIN INSENSITIVE*)/-fold hydrolase, which jointly perceives karrikins (smoke-derived butenolides), were made, and *KAI2* and *MAX2* function. Consequently, these findings imply that association analysis in conjunction with transcriptome analysis is a potent strategy for identifying target genes involved in rapeseed root growth.

Weighted gene co-expression network analysis (WGCNA) is a helpful method for analyzing transcriptome data to discover the network of genes linked with specialized roles and even discover new genes (Lou et al., 2017). Three large modules (green-yellow, blue, and magenta) made up of strongly linked genes were detected in this study utilizing WGCNA and all 49,080 DEGs (**Figure 3A**). The genes of the three modules (green-yellow, blue, and magenta) were highly significantly enriched into thirteen pathways, including the mRNA surveillance pathway, pentose phosphate pathway, N-Glycan biosynthesis, ether lipid metabolism, phosphonate and phosphinate metabolism, pyrimidine metabolism, fructose, and mannose metabolism, and glyoxylate and dicarboxylate metabolism, according to the Kyoto Encyclopedia of Genes and Genomes (KEGG) analysis (**Tables S15–S17**). The metabolism of fructose and mannose was regulated by four DEGs from the green-yellow module: *BnaA09G0693700ZS, BnaA09G0715400ZS*, *BnaA02G0312100ZS, BnaA02G0213400ZS*, and *BnaA03G0520200ZS*. One of the main sugars in plants, fructose plays a different role in plant growth than the other two main sugars, sucrose, and glucose. The t*pst* mutations in Arabidopsis cause fructose sensitivity. Fructose, as opposed to sucrose and glucose, slows down the growth of the primary roots in a variety of Arabidopsis ecotypes when present in small amounts (Zhong et al., 2020). IAA is one of the tryptophan-derived signals known as auxins, which play a role in the majority of plant growth processes (Woodward and Bartel, 2005). The blue module DEGs *BnaC07G0541500ZS, BnaA06G0306800ZS*, and *BnaC03G0581900ZS* were engaged in tryptophan metabolism. Serotonin, a chemical generated from tryptophan, controls root growth likely by functioning as an endogenous auxin inhibitor (Pelagio-Flores et al., 2011). Similarly, N-Glycan biosynthesis was mediated by four DEGs in the magenta module, *BnaC03G0226600ZS, BnaC06G0444300ZS, BnaA02G0056500ZS*, and *BnaC06G0057400ZS* (**Table S16**). According to Frank et al. (2021), complex-type N-glycosylation deficiency alters the way plants react to exogenous phytohormones, causing root-hair elongation to occur earlier and more frequently.

**Figure 5:**
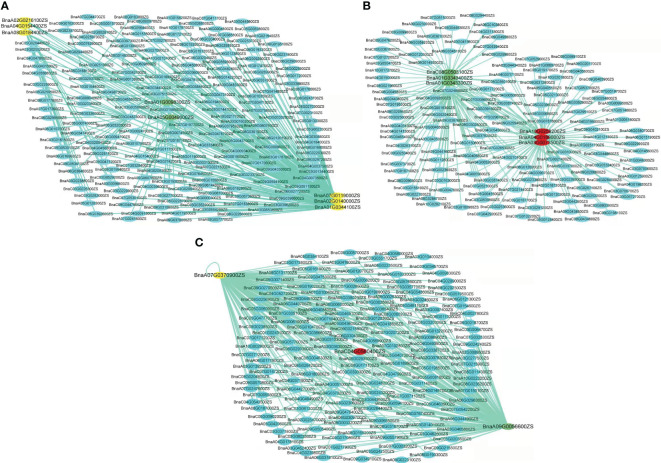
Networks of genes in turquoise, blue, and yellow modules. **(A–C)** Correlation of networks in turquoise, blue, and yellow modules, respectively. The yellow color in the network indicates the candidate genes overlapped by GWAS and WGCNA, the green color indicates the candidate genes overlapped by GWAS and DEGs, and the red color indicates genes overlapped by GWAS, WGCNA, and DEGs.

Conversely, the turquoise module’s six out of the seven WGCNA/GWAS overlapped genes (*BnaA01G0344100ZS, BnaA02G0140000ZS, BnaA02G0216100ZS, BnaA04G0154400ZS, BnaA07G0119000ZS*, and *BnaA08G0184400ZS*) showed some sporadic relationships to one another (**Figure 5A**; **Table 2**). A homolog of Arabidopsis *PIN-FORMED 7* (*AtPIN7*), *BnaA07G0119000ZS*, was found to influence root growth by regulating the radial growth of root systems (Rosquete et al., 2018). Moreover, it has been noted that the gene *BnaA02G0140000ZS*, which codes for the Arabidopsis *CYTOKININ RESPONSE FACTOR 3* (*AtCRF3*), controls the onset of LR (Jeon et al., 2016).

Furthermore, it has been revealed that GWAS, WGCNA, and differential expression analysis combined is an effective technique to find key genes in rapeseed (Ahmad et al., 2022), maize (Li P. et al., 2021), and Rice (Li et al., 2017). Moreover, Li P. et al. (2021) discovered four DEGs by combining GWAS, WGCNA, and differential expression analysis in rapeseed; correspondingly, our DEG, WGCNA, and GWAS analysis discovered four DEGs in the blue and yellow modules—*BnaC04G0560400ZS, BnaA03G0073500ZS, BnaA04G0189000ZS*, and *BnaA10G0258200ZS*—that were highly expressed in LK1T2, LK2T2, and HK1T2 (**Table S3**; **Figures 5B, C**). The ortholog of the *Glyceraldehyde-3-phosphate dehydrogenase* (*GAPA2*) found in Arabidopsis, *BnaC08G0503100ZS*, is implicated in the metabolism of glucose, which has been shown to influence various root directional responses such as root waving and coiling and result in impaired root architecture (Singh et al., 2014). Similarly, *CAX1* functions in cellular manganese ion homeostasis and is homologous to *BnaC04G0560400ZS*. Yang et al. (2008) found that the structure and characteristics of the root epidermal cells had significantly changed when Mn deficiency was applied to Arabidopsis seedlings. When plants were cultivated at a light intensity of >50 μmol m^−2^ s^−1^ with manganese present, root hair elongation was severely impeded, but Mn-deficient seedlings displayed improved root hair development. Also, the circadian rhythm response is mediated by *BnaA03G0073500ZS*, an Arabidopsis *REEILLE1* (*RVE1*) homolog. The model plant *Arabidopsis thaliana* deep root contains a cluster of stem cells that develop into LRs, which cause the overlying root tissues to give way to the formation of new organs. The circadian clock is reset throughout LR development, enabling auxin signaling to be regulated to facilitate organ formation (Voß et al., 2015). This research will enable us to pinpoint the genes and biological processes involved in the architecture of rapeseed roots. More interestingly, this research might reveal candidate genes that would support molecular breeding and genetic engineering initiatives aimed at rapeseed root development enhancement.

Growth signals induce the regulation of the transcription of genes involved in development as well as several biological functions, notably the metabolism of carbohydrates, amino acids, and lipids. We surmised that the genes identified to be corresponding to the root growth and KUE genes in *A. thaliana* might be closely associated with the root growth and KUE in *B. napus*. It may be possible to confirm the function of these genes in root development and KUE in *B. napus* through additional study and gene validation. These possible DEGs provide the framework for a more thorough analysis of the potassium stress response pathways in *B. napus*.

## Conclusion

In summary, this research demonstrates the effectiveness of the DEG technique for identifying significant genes in the two contrasting rapeseed lines and provides an extensive transcriptome analysis of rapeseed roots between two contrasting genotypes in response to potassium absorption efficiency. The development of rapeseed roots in response to potassium absorption efficiency was shown to be linked with the DEGs engaged in carbohydrate, lipid, and amino acid metabolisms, hormones, and regulatory networks. These findings shed important light on the molecular mechanisms underlying broad transcriptional levels of root growth. In addition to providing thorough and precise data for identifying the potassium absorption efficiency of candidate rapeseed, our big transcriptome dataset results also identified candidate genes responsible for variations in rapeseed root growth. To achieve potassium absorption efficiency and yield quality, genetic modifications in these candidates may be crucial.

## Data availability statement

The data presented in the study are deposited in the National Center for Biotechnology Information Sequence Read Archive (http://www.ncbi.nlm.nih.gov/sra/), accession number PRJNA949847.

## Author contributions

The research was designed and coordinated by all authors. LK, ZT, and XW provided the experimental materials and supervised the experiment. NA conducted the root studies. KL and SBS performed the data analysis. SMT edited the manuscript. SI experimented and wrote the manuscript. The manuscript was modified with help from XD and HW. All authors contributed to the article and approved the submitted version.
