# Supplementary material for: Transcriptome analysis reveals key regulatory genes for root growth related to potassium utilization efficiency in rapeseed (Brassica napus L.)
Source: Front Plant Sci. 2023 Jul 19;14:1194914. doi: 10.3389/fpls.2023.1194914 (PMC10400329; doi:10.3389/fpls.2023.1194914)
Supplement: Supplementary file 1 [file DataSheet_1.zip › Supplementary File 2.docx]

**Transcriptome Analysis Reveals Key Regulatory Genes for Root Growth Related to Potassium Utilization Efficiency in Rapeseed (*Brassica napus* L.)**

**Sani Ibrahim, Nazir Ahmad, Lieqiong Kuang, Keqi Li, Ze Tian, Salisu Bello Sadau, Sani Muhammad Tajo, Xinfa Wang, Hanzhong Wang and Xiaoling Dun**

*** Correspondence:** Xiaoling Dun: [dunxiaoling@caas.cn](mailto:dunxiaoling@caas.cn)


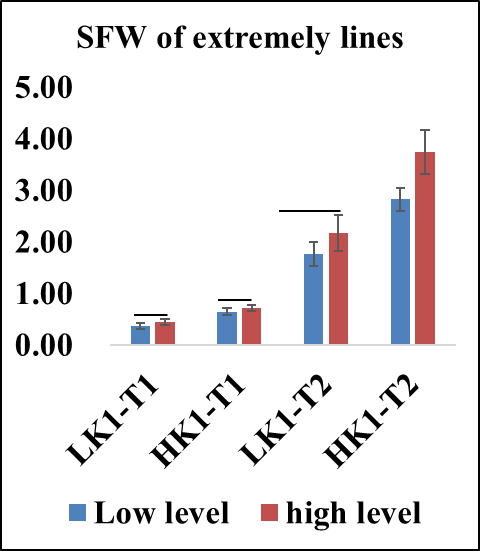

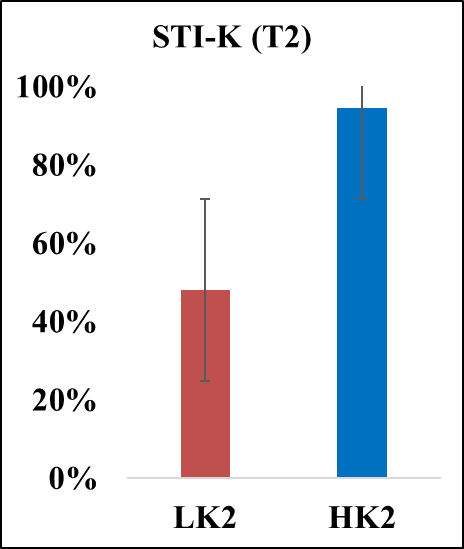


**

**

**

**

**

**(a)**

**(b)**

**

**

*

**

**

**Supplementary Figure 1:** Potassium absorption potential across the groups. (a) Groups with low and high SFW under both high and low K levels at two time points (7 DAT & 14 DAT). (b) Groups with low and high STI-K (The ratio of SFW-LK/SFW-HK at 14 DAT


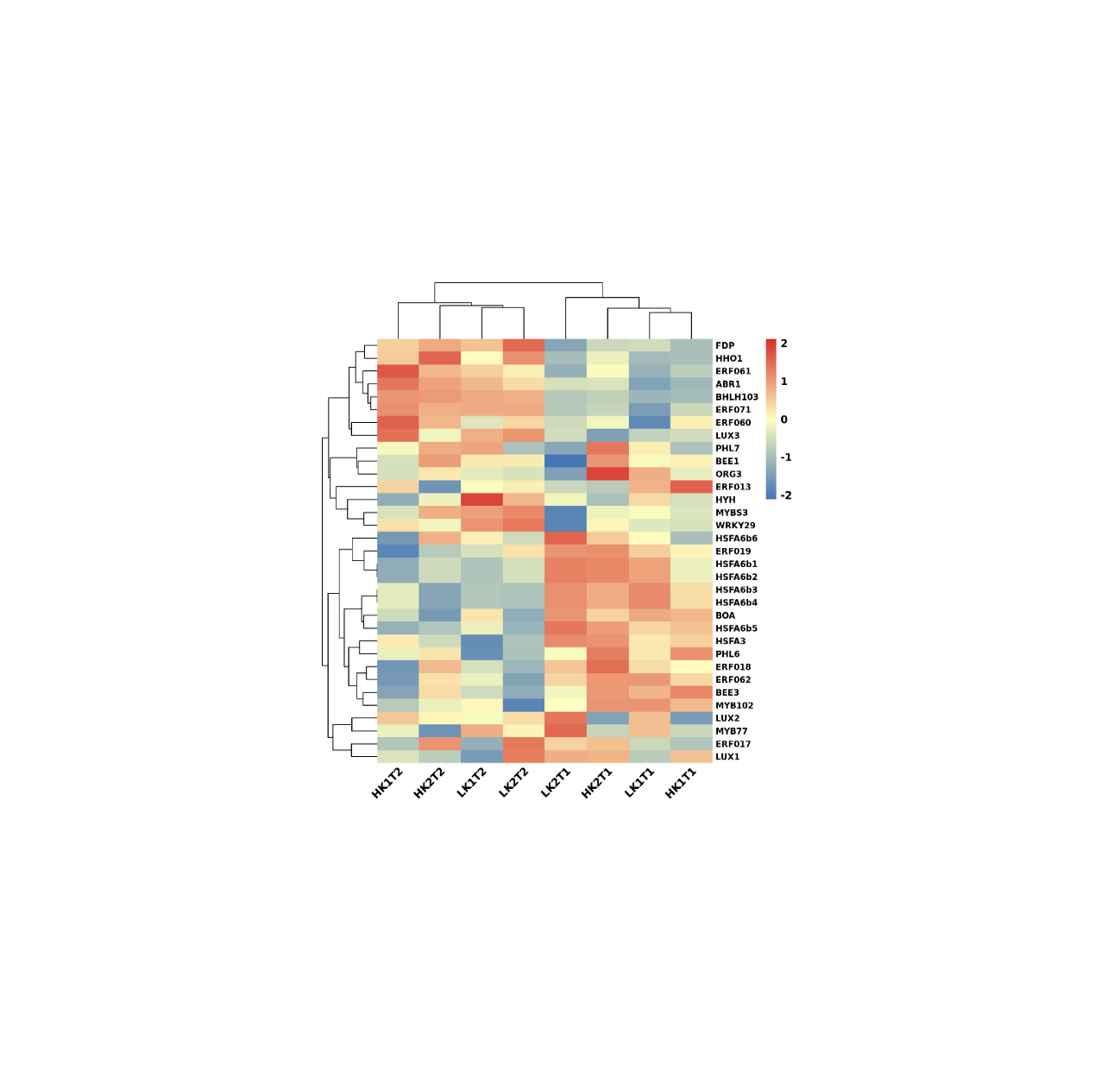

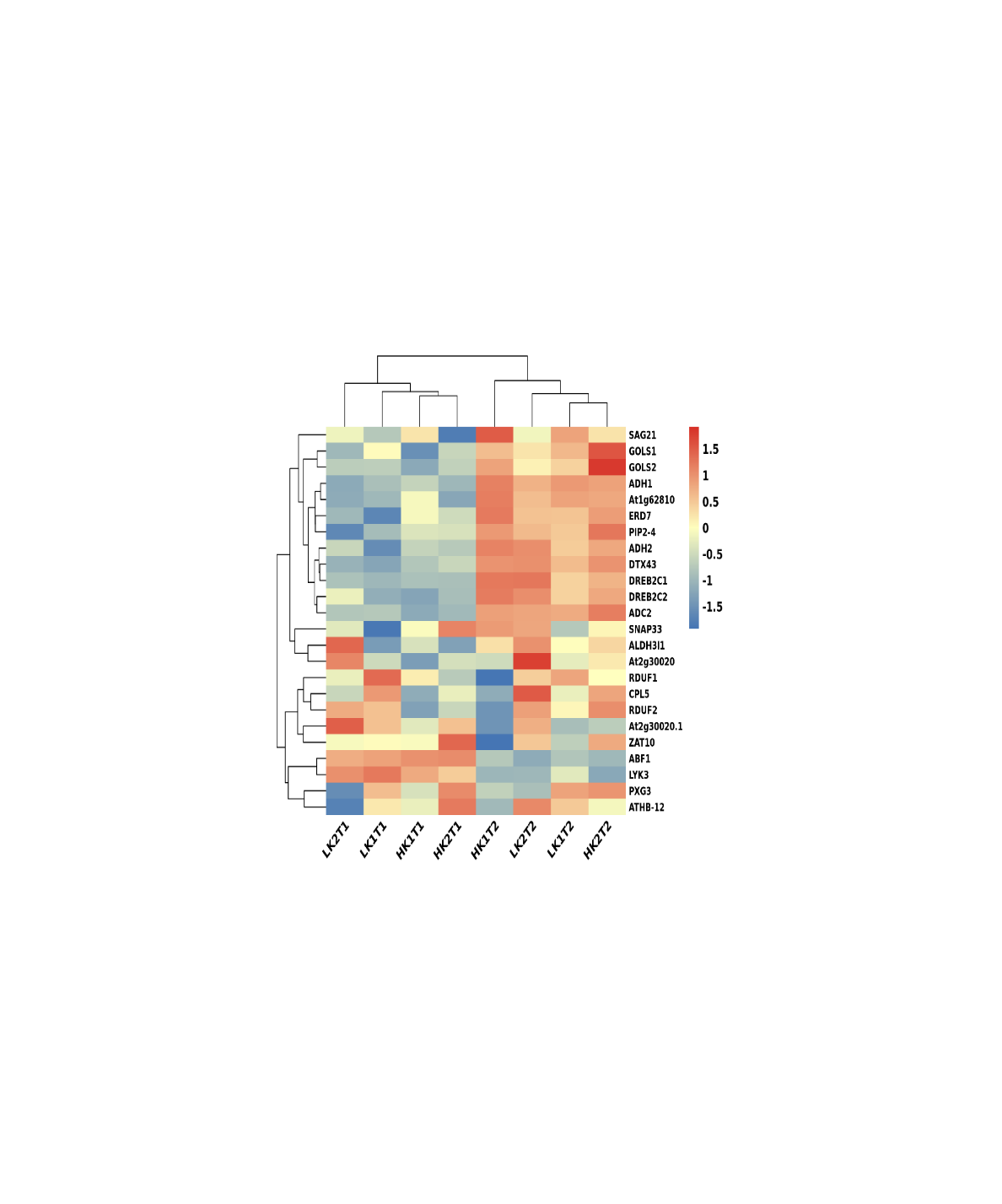


**Transcription factors**

**(a)**

**Abscisic acid**

**(b)**


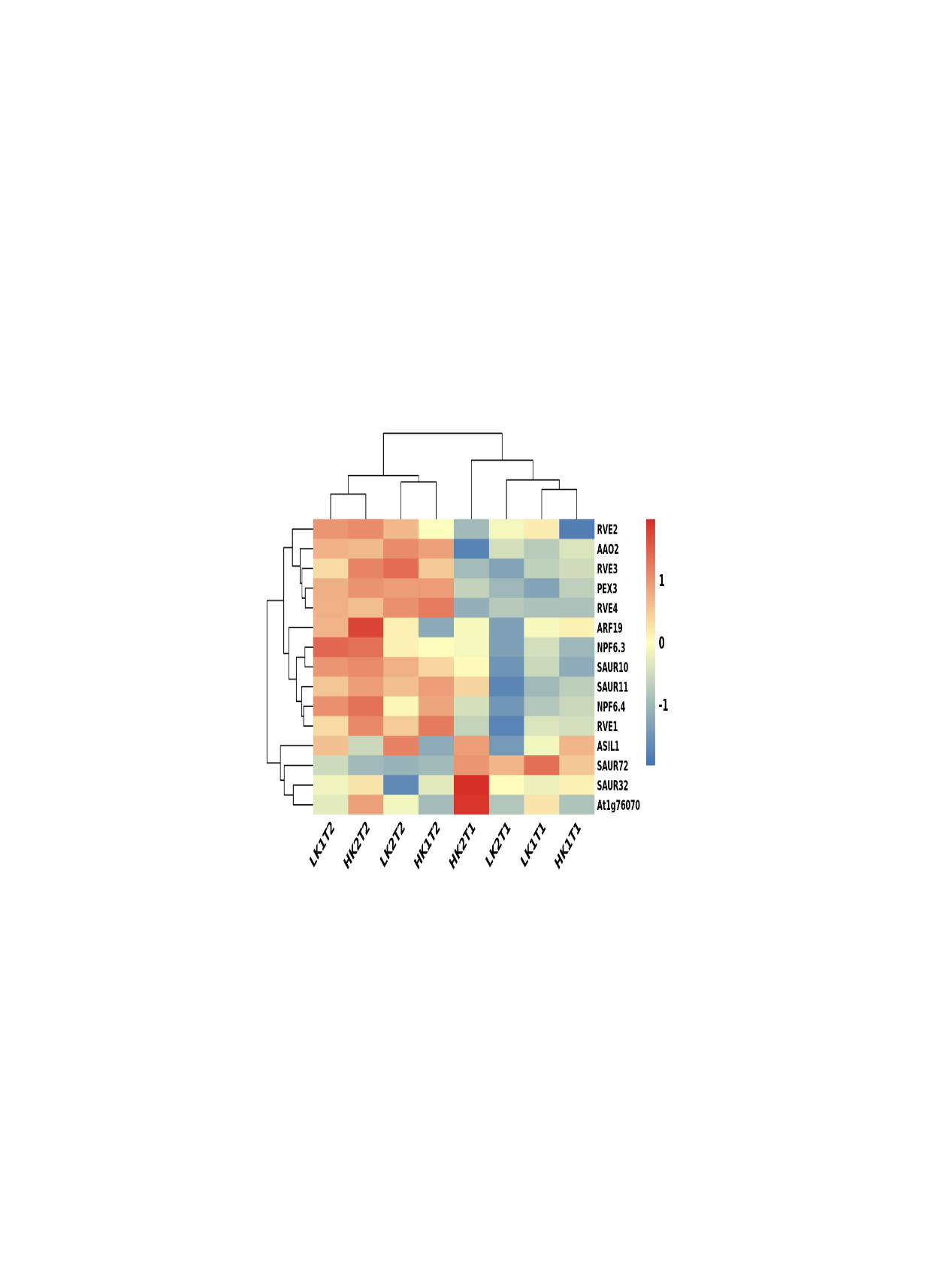


**Auxin**

**(c)**


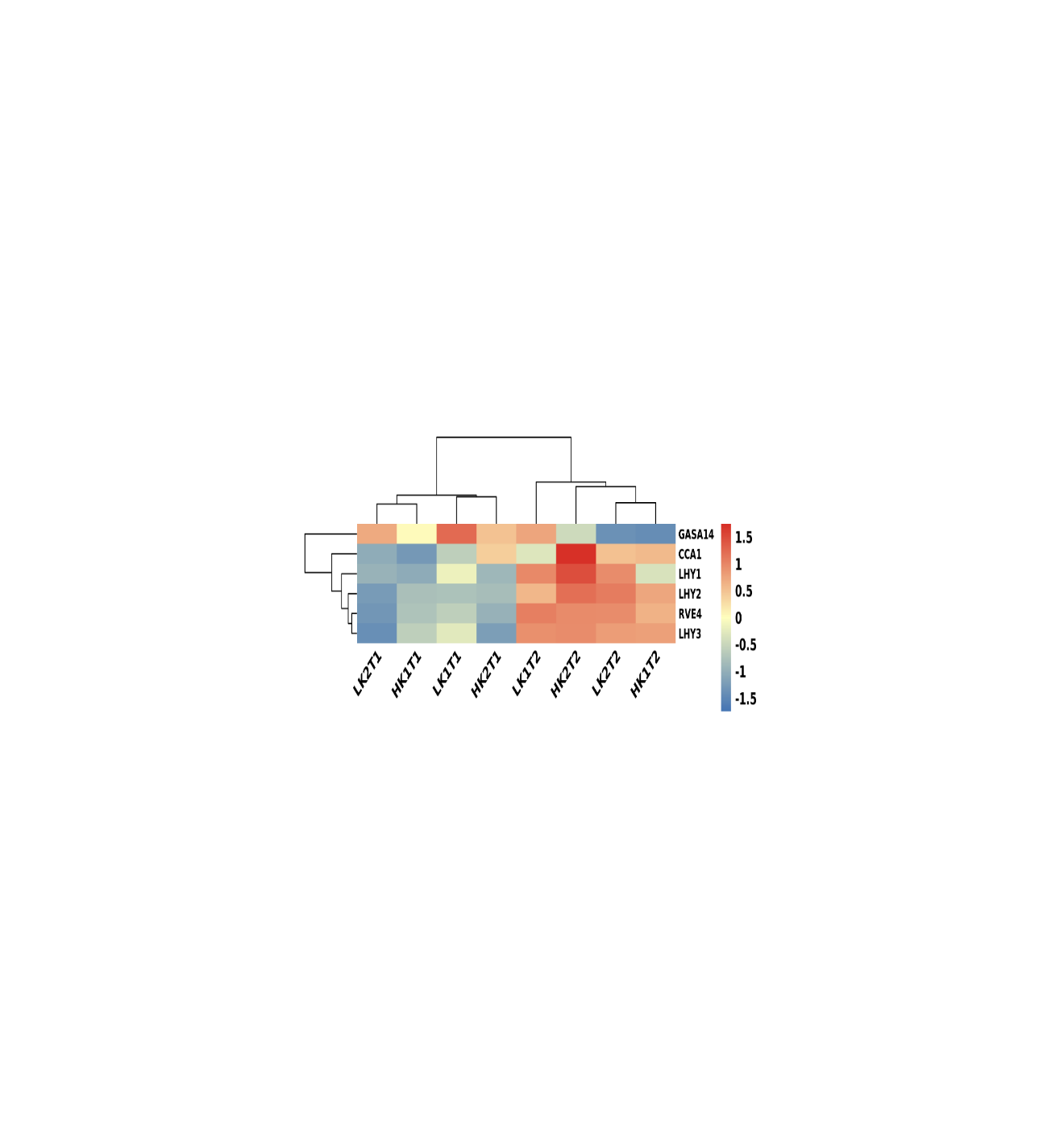


**Gibberellic acid**

**(d)**


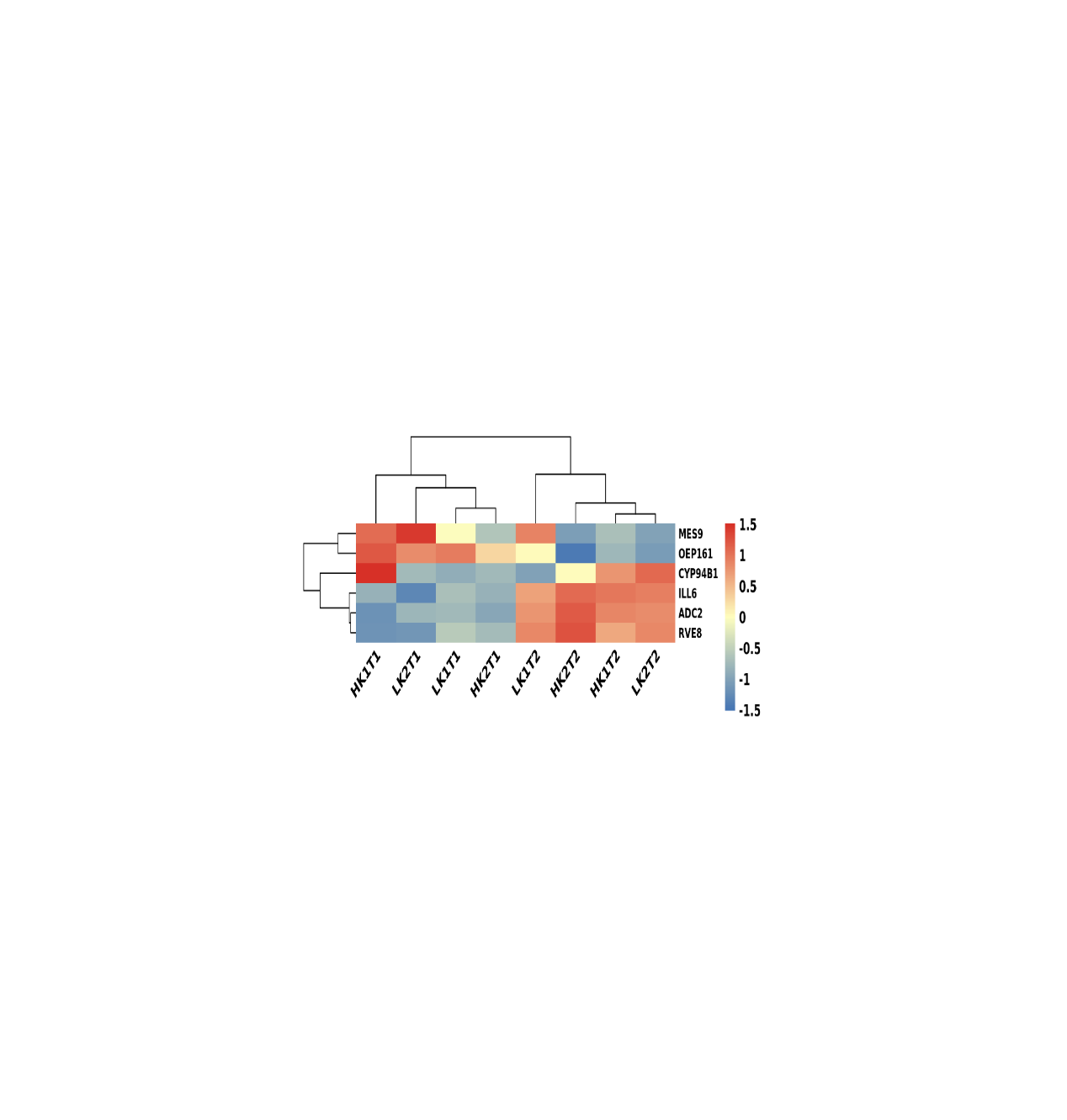


**Jasmonic acid**

**(e)**


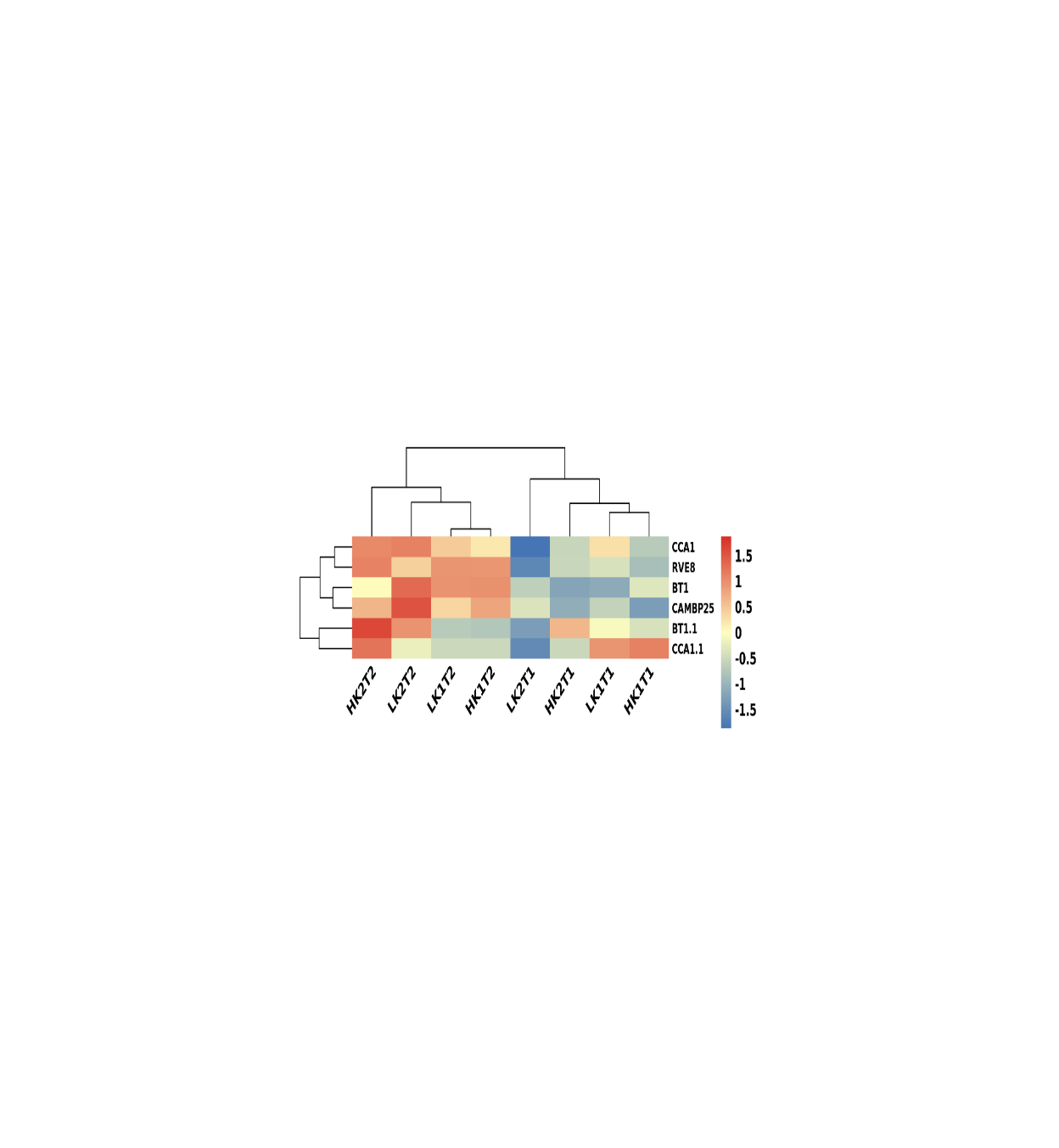


**Jasmonic acid**

**(f)**

**Supplementary Figure 2. a, b, c, d, e & f,** Hierarchical cluster analysis of DEG-related transcription factors and phytohormones signaling pathways. The red color represents the expression of up-regulated genes, and the blue color represents down-regulated genes using normalized FPKM


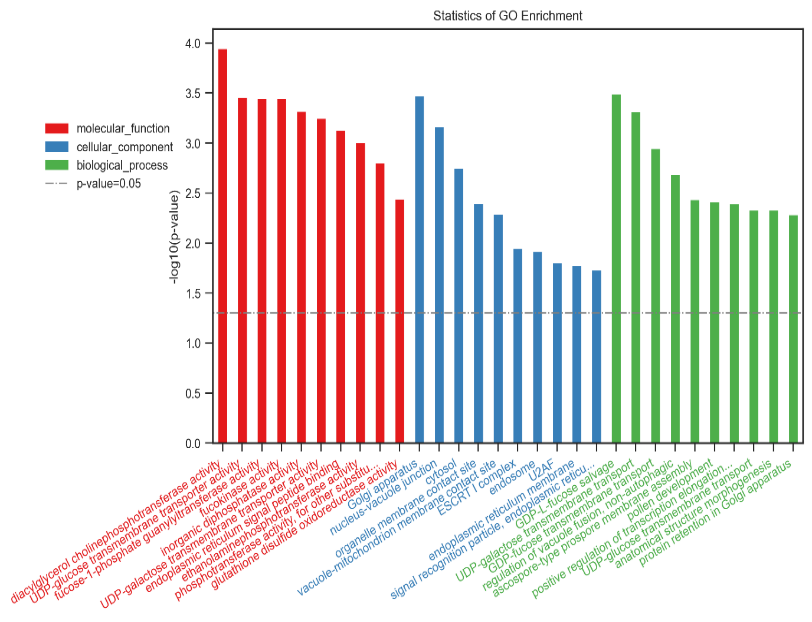

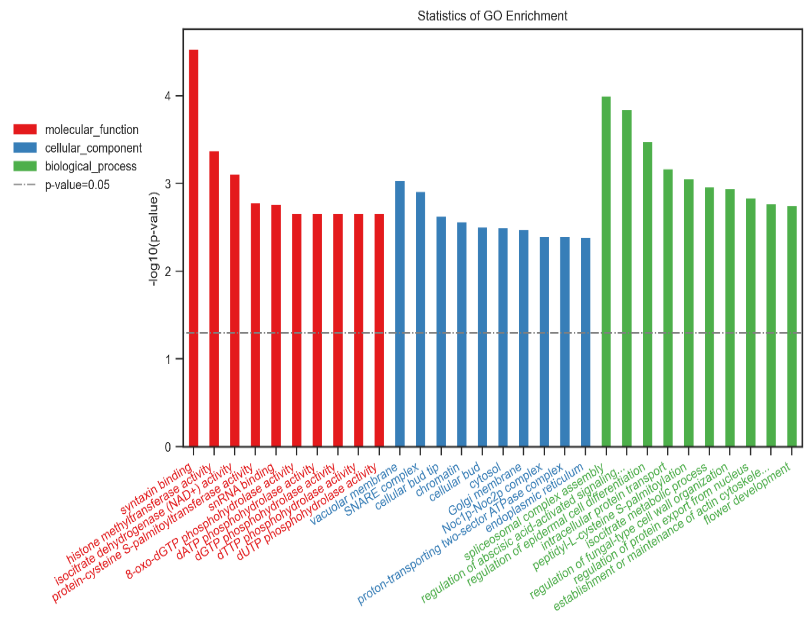

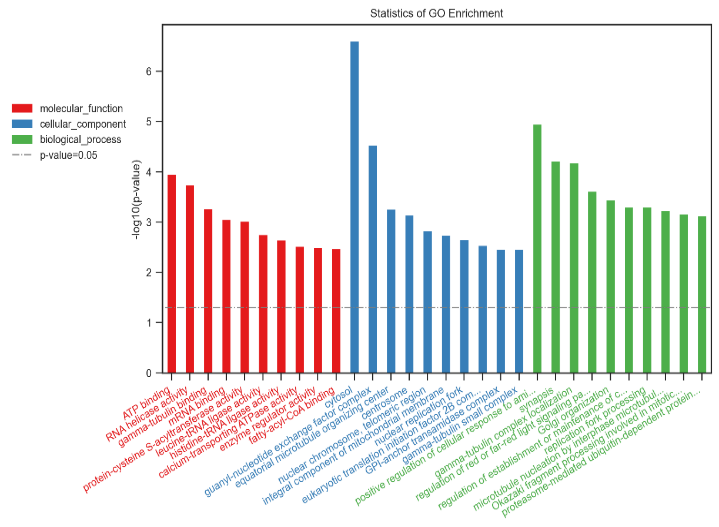


**(a)**

**(b)**

**(c)**

**Supplementar**y **Figure 3.** Functional annotation of all the modules detected DEGs. **(a, b & c)** Gene Ontology (GO) analyses of the differentially expressed genes in the greenyellow, black and magenta modules, respectively.


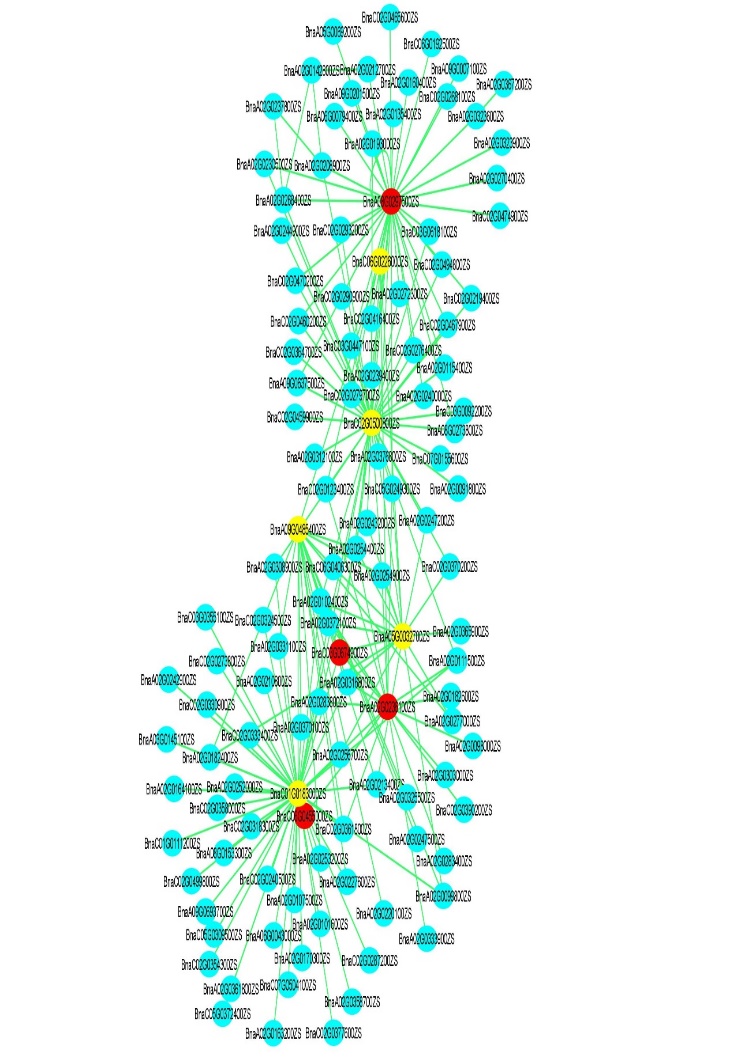

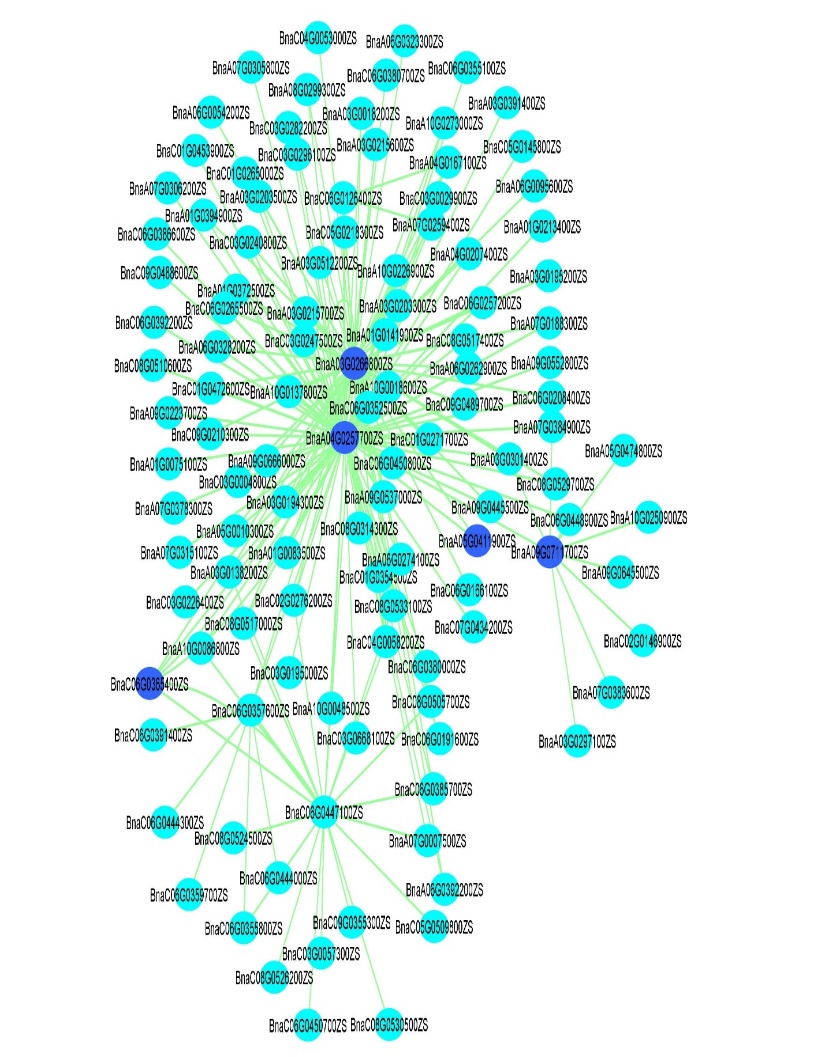


**(a)**

**(b)**

**Supplementary Figure 4.** Networks of genes in green-yellow and magenta modules. **(a and b)** Correlation of networks in green-yellow and magenta modules, respectively.
